# Video Games as a Means to Reduce Age-Related Cognitive Decline: Attitudes, Compliance, and Effectiveness

**DOI:** 10.3389/fpsyg.2013.00031

**Published:** 2013-02-01

**Authors:** Walter R. Boot, Michael Champion, Daniel P. Blakely, Timothy Wright, Dustin J. Souders, Neil Charness

**Affiliations:** ^1^Department of Psychology, Florida State UniversityTallahassee, FL, USA; ^2^Cognitive Science and Engineering, Technological Entrepreneurship and Innovation Management, Arizona State UniversityMesa, AZ, USA

**Keywords:** cognitive training, video games, transfer of training

## Abstract

Recent research has demonstrated broad benefits of video game play to perceptual and cognitive abilities. These broad improvements suggest that video game-based cognitive interventions may be ideal to combat the many perceptual and cognitive declines associated with advancing age. Furthermore, game interventions have the potential to induce higher rates of intervention compliance compared to other cognitive interventions as they are assumed to be inherently enjoyable and motivating. We explored these issues in an intervention that tested the ability of an action game and a “brain fitness” game to improve a variety of abilities. Cognitive abilities did not significantly improve, suggesting caution when recommending video game interventions as a means to reduce the effects of cognitive aging. However, the game expected to produce the largest benefit based on previous literature (an action game) induced the lowest intervention compliance. We explain this low compliance by participants’ ratings of the action game as less enjoyable and by their prediction that training would have few meaningful benefits. Despite null cognitive results, data provide valuable insights into the types of video games older adults are willing to play and why.

## Introduction

As we age, we can expect to experience greater difficulty with tasks involving a number of perceptual and cognitive abilities (e.g., Schaie, 1996; Salthouse, 2010). These declines are associated with decreased ability to perform the everyday tasks required for functional independence, such as the ability to drive a car, adhere to a medication schedule, and manage finances (e.g., Ball et al., 1993; Diehl et al., 1995; Royall et al., 2004). An important question is whether age-related cognitive and perceptual declines can be slowed or reversed (Hertzog et al., 2009; Lövdén et al., 2010).

Two challenges must be overcome in the development of effective cognitive aging interventions. First, over a century of research suggests that training gains are often extremely specific (Boot and Blakely, 2011). Training on one task almost invariably results in improvement, but this improvement rarely transfers to novel tasks or even tasks similar to the trained task. However, in studies involving young adults, action video game training appears to improve a broad range abilities (e.g., Green and Bavelier, 2006a,b, 2007; Li et al., 2009, 2010; Chisholm et al., 2010; Colzato et al., 2010; Granek et al., 2010; Green et al., 2010, 2012; Clark et al., 2011; but see also Boot et al., 2011). These results are remarkable because (1) transfer assessment tasks were dissimilar from the trained games, (2) improvements were observed in abilities that show large age-related decline, and (3) improvements were often engendered after a short period of training (10–50 h).

The second challenge to overcome is designing interventions that encourage intervention compliance. Interventions that include video games would seem to be ideal to encourage compliance as video games are assumed to be inherently motivating and enjoyable. However, game designers often do not consider the older adult demographic in their design and marketing of games, and the types of games that appeal to older adults may be very different from the games that appeal to younger adults. Furthermore, there may be a mismatch between the games that older adults enjoy playing and the types of games that result in the largest perceptual and cognitive gains. Older adults report a preference for games that involve intellectual challenge compared to the fast-paced action games that tend to produce the broadest transfer of training (Pearce, 2008; Nap et al., 2009; McKay and Maki, 2010). However, even games that promote intellectual challenge may not be effective in inducing compliance. Ackerman et al. (2010) asked participants who had just completed an intervention involving the brain fitness game Big Brain Academy^®^ whether or not they planned on ever playing the game again. Sixty-three percent of participants indicated that they did not.

The current study aimed to assess the efficacy of game interventions in improving cognition. In addition, and potentially just as important, the current study investigated the factors that shape motivation and compliance with respect to game-based interventions in an older adult sample and evaluated older adults’ attitudes and expectations with respect to video game interventions. One game was an action game because these types of games have been previously reported to be effective at improving a host of abilities. The other was a brain fitness game similar in style to a previous game found to be ineffective (Ackerman et al., 2010), but contained features of games that seniors typically enjoy. We were particularly interested in handheld devices as a means to deliver training since these have the advantage of being relatively cheap, easy to use, and portable compared to interventions delivered on a personal computer or gaming console. However, these advantages would need to overcome usability issues that might be associated with small screens and difficult-to-use input devices (see Boot et al., 2012 for more discussion).

## Materials and Methods

### Participants

Sixty-two participants (Mean Age = 74 years old, SD = 6, range = 54–86) were recruited from the Tallahassee community and assigned to one of two game intervention conditions or a no-contact control group (Table 2). Participants lived in independent living situations, were Caucasian, received a minimum score of 25 on the MMSE (*M* = 29, SD = 1.04), and most (90%) were retired. Pre-screening ensured participants had an “intact” score according to the Short Portable Mental Status Questionnaire (less than or equal to two errors; Pfeiffer, 1975), and demonstrated no significant memory deficits using the Wechsler Memory Scale (Logical Memory subscale; age-adjusted criterion; Wechsler, 1997). This pre-screening helped to ensure that participants were neurologically intact; otherwise participants were not screened based on medication use or neurological function or disease. Average near visual acuity was 20/32. Participants were paid 10 dollars an hour for all laboratory visits. All procedures were approved by Florida State University’s Human Subjects Committee, and written informed consent was obtained from all participants.

### Study design

With the exception that spouses/partners were assigned to the same condition, participants were randomly assigned to one of three groups. One group received an action video game to play, another group received a “brain fitness” game to play, and the third group served as a control group for test-retest effects. A battery of ability measures was administered once before and once at the end of the study to assess any potential change as a result of gameplay over the course of three 1.5 to 2-h sessions before and after a 12-week period.

#### Cognitive assessment battery

Assessment measures fell into one of four broad categories: Perceptual Speed, Memory, Selective Attention/Executive Control, and Reasoning Ability (Table 1). Well-being was also assessed before and after training. Full details of each task can be found at: http://walterboot.net/GameStudy/DetailedMethods.pdf. Here we present a brief overview of each measure.

**Table 1:** **List of principal cognitive outcome measures**.

Task name	Construct assessed	Critical measure	Number of test trials/questions	Comments
Simple/complex RT	Processing speed	Reaction time	80	Based on Czaja et al. (2006)
Number comparison	Processing speed	Accuracy (timed)	96	Ekstrom et al. (1976)
Visual search	Processing speed	Accuracy	72	Based on Sekuler and Ball (1986)
Corsi block tapping	Spatial memory	Accuracy	24	Based on Corsi (1972)
Everyday recognition	Memory	Accuracy	15	Modification of Allaire and Marsiske (1999)
Meaningful memory	Memory	Accuracy	20	Hakstian and Cattell (1975)
MSEQ	Memory	Confidence	20	West et al. (2005)
Flanker task	Selective attention	Flanker interference	80	Based on Eriksen and Eriksen (1974)
Task switching	Executive control	Switch cost	90	Based on Basak et al. (2008)
Raven’s matrices	Reasoning	Accuracy (timed)	18	Modification of Raven et al. (2003)
Everyday reasoning	Reasoning	Accuracy	21	Modification of Allaire and Marsiske (1999)
Letter sets	Reasoning	Accuracy (timed)	30	Ekstrom et al. (1976)
MIDUS	Well-being	Well-being ratings	42	Brim et al. (1996)

**Table 2:** **Demographics for all participants and for participants who completed the study as a function of group assignment**.

	*N*		Mean age		Proportion male	
	All	Completed	All	Completed	All	Completed
Control	20	20	72 (1.4)	72 (1.4)	0.45	0.45
Brain fitness game	21	20	74 (1.2)	73 (1.1)	0.33	0.35
Action game	21	14	75 (1.5)	73 (1.9)	0.48	0.50

*Standard errors listed within parenthesis*.

*For the game groups completion rates favored Brain Fitness: *X*^2^(1) = 5.56, *p* < 0.02*.

##### Processing speed

*Simple and choice reaction time* Participants saw a square appear at the center of the screen and were asked to respond quickly when they saw it (simple RT), or pushed one of two keys depending on which side of the screen the square appeared on (choice RT).

*Number comparison* Participants had to judge as quickly as possible whether the two strings of numbers were the same or different. The same form was used pre and post-test. Responses were indicated by writing or not writing a mark between the two number strings using a pen.

*Visual search* Participants viewed a briefly presented search display. Distractors were square items, and the target was a triangle within a circle. After the search display appeared it was masked, and participants were asked to indicate where the target appeared.

##### Memory

*Corsi block tapping* Participants viewed computer images with a number of squares that turned red, then back to gray one at a time. Participants were asked to remember the sequence of color changes, and to click using the mouse each square in the same order in which they changed. Sequences varied from four to seven color changes.

*Everyday recognition* Participants were given stimuli such as banking statements and prescription labels to remember. They had 1 min to memorize these materials, and 1 min to answer questions about the memorized materials. Two forms were created by dividing the Everyday Cognition Battery (ECB) Recognition Questionnaire into two. One form was administered before training and one after training, with the order of forms counterbalanced across participants.

*Meaningful memory* Participants were given a list of 20 nouns and words that described each noun and had 1 min and 15 s to memorize this information. Ten minutes later, they were given the same nouns, and a choice of four descriptors, none of which matched the original descriptor exactly. The task of the participant was to pick the word closest in meaning to the original descriptor paired with each noun. The same form was used pre and post-test.

*Memory Self-Efficacy Questionnaire* Participants were presented with a number of scenarios varying in difficulty and were asked to rate their confidence that they could perform the memory tasks described (from 0% confidence, to 100% confidence). Of primary interest was self-confidence of memory ability. The same form was used pre and post-test.

##### Selective attention/executive control

*Flanker task* Participants saw an arrow at the center of the screen and had to respond to whether the arrow pointed to the left or right. Two arrows appeared to either side of the target arrow and could be either congruent or incongruent with the target arrow (pointing in the same or different direction). Of primary interest was flanker interference, or the cost associated with the flanking arrows providing incongruous information. This is thought to reflect a failure of selective attention, or inability to restrict processing to relevant information while excluding the processing of irrelevant information.

*Task switching* Participants viewed sequences of numbers and judged whether numbers were high or low, or odd or even by pushing one of two keys as quickly as possible. The color of the screen informed participants which task to perform. The task to be performed was unpredictable. Switch costs were calculated to reflect the cost in terms of speed and accuracy of having to switch from one task to the other^1^.

##### Reasoning ability

*Raven’s matrices* The Raven’s Advanced Matrices test was divided into two forms of approximately equal difficulty (18 questions each). Order of administration was counterbalanced across participants. Each trial presented participants a visual pattern with a piece cut out of it, and eight options to fill in the missing piece (one being correct).

*Everyday reasoning* Participants were given stimuli such as different nutrition labels or bank statements and were asked to answer questions about them. Two forms were created by dividing the ECB Reasoning Questionnaire into two. One form was administered before training, and one after training, with the order of forms counterbalanced across participants.

*Letter sets* Participants viewed sets of letters with all but one letter set being governed by a common rule. The task of the participant was to discover the rule and mark the letter set that did not follow the rule. The same form was used pre and post-test.

##### Well-being

*Midlife in the United States Scale* This survey asked participants to rate their well-being. The Midlife in the United States Scale (MIDUS) has subscales of well-being focusing on autonomy, environmental mastery, positive relationships with others, personal growth, life purpose, and self-acceptance.

*Game perception and attitude surveys* In addition to the cognitive assessment battery, participants who received a game to play were also asked to complete two surveys, one which assessed their attitudes toward the game they were given to play and one which assessed their belief that the game they were given to play was capable of improving perceptual and cognitive abilities^2^.

*Survey and phone data* Participants who received a game to play were given a diary in which they were asked to keep a record of their game play (date and amount of time played). They were also encouraged to make notes about their game experience. Phone calls were placed every 1–2 weeks to each participant in the game groups. These calls asked participants about their gameplay frequency. These data served as measures of intervention compliance.

*Game training* The *Nintendo DS*^™^ *Lite* gaming system was used to deliver the video game intervention. Participants who were assigned to one of the game groups were given a brief tutorial and demonstration of their training game before they left the laboratory on the last day of the pre-training cognitive assessment battery. Participants were requested to play their assigned game five times a week, for 1 h each gaming session. In total, participants should have obtained 60 h of game experience over the course of the study.

The Action Game group received the racing game *Mario Kart DS*^®^. In this game, the player races against other computer-controlled characters while avoiding dangers on the race track and using items and weapons against opponents. *Mario Kart DS*^®^ was chosen based on past research demonstrating that action game training can produce a variety of benefits. Although these previous studies have mostly used violent first-person shooters, older adults tend to dislike this type of game experience (Nap et al., 2009). Non-violent games with less realistic cartoon depictions, like *Mario Kart DS*^®^, have been found to be more acceptable to older adults (McKay and Maki, 2010). Despite differing significantly from first-person shooters, *Mario Kart DS*^®^ shares many characteristics of an action game, with action games being defined as games “that have fast motion, require vigilant monitoring of the visual periphery, and often require the simultaneous tracking of multiple targets” (e.g., Green and Bavelier, 2006a, p. 1466). Racing success requires players to monitor multiple fast-moving racers that can attack the player with various traps and weapons, and who the player can attack to take the lead. Attention must also be divided between two different screens, one depicting an ego-centric perspective and one showing a birds-eye view of the race. Monitoring of multiple locations and multiple enemies is consistent with first-person shooters. However, “monitoring of the visual periphery” may be somewhat minimized given the size of the game screens.

The Brain Fitness group received *Brain Age 2*^™^, a brain-training game largely targeted to older adults as a means to improve cognitive performance. Players engage in a multitude of activities emphasizing memory, reaction time, language, and mathematical ability. For most activities, the *Nintendo DS*^™^ is held like a book and the stylus is used to input letters, numbers, or mathematical operators depending on the nature of the activity. Some activities used voice recognition. *Brain Age 2*^™^ was chosen because of its explicit focus on cognitive training, although previous research has found similar training activities to produce no effect on cognition (Ackerman et al., 2010; Owen et al., 2010; but see more recently Nouchi et al., 2012).

Finally, one group received no training to control for test-retest effects. Perceptual and cognitive abilities of this group were tested, and were tested again after approximately 3 months.

## Results

First, we turn our attention to whether either video game intervention had a significant effect on cognition, then we discuss issues of compliance, and finally we consider perceptions and attitudes toward game interventions. Fifty-four of 62 participants completed the study. Of the participants who did not complete the study, one was assigned to the Brain Fitness group and seven were participants assigned to the Action Game Group. This differential attrition was the first indication that although we predicted the action game to be more effective at improving cognition, older adults would show a preference for the brain fitness game.

### Cognitive battery

Due to computer error, misadministration of an assessment task, or participants skipping answers or otherwise not providing a complete data set, some participants had to be excluded from analysis of individual tasks. Improvement scores were computed by comparing pre-training and post-training performance (such that positive scores always corresponded to greater improvement). Of primary interest was whether a significant effect of group (Control, Action Game, Brain Fitness Game) was observed. Table 3 lists means and standard errors for each task as a function of time (pre, post-training) and group. Reaction time measures included only accurate trials. First, an ANOVA approach was taken looking for group differences in each individual task. This approach revealed no greater improvement for either game group (Action Game or Brain Fitness Game) relative to the no-game control group^3^.

**Table 3:** **Pre and post-training scores**.

		Control		Brain fitness		Action game	
		Pre	Post	Pre	Post	Pre	Post
Simple/choice RT (*n*_c_ = 20, *n*_BF_ = 20, *n*_AG_ = 14)	Simple RT (ms)	365 (15)	351 (10)	359 (12)	357 (12)	352 (13)	342 (18)
	Complex RT (ms)	396 (14)	394 (13)	414 (10)	427 (17)	397 (14)	398 (13)
	Simple accuracy	0.96 (0.02)	0.97 (0.02)	0.98 (0.01)	0.99 (0.01)	0.97 (0.02)	0.97 (0.02)
	Complex accuracy	0.97 (0.01)	0.96 (0.01)	0.98 (0.01)	0.98 (0.01)	0.95 (0.04)	0.98 (0.01)
Number comparison (*n*_c_ = 20, *n*_BF_ = 20, *n*_AG_ = 14)		38.20 (2.40)	38.85 (2.43)	37.75 (2.40)	39.40 (2.43)	43.07 (2.97)	41.57 (2.90)
Visual search (*n*_c_ = 20, *n*_BF_ = 20, *n*_AG_ = 14)	Near	0.19 (0.02)	0.25 (0.04)	0.29 (0.06)	0.27 (0.06)	0.31 (0.07)	0.34 (0.07)
	Middle	0.19 (0.02)	0.20 (0.03)	0.24 (0.05)	0.26 (0.05)	0.23 (0.05)	0.27 (0.07)
	Far	0.15 (0.02)	0.18 (0.02)	0.21 (0.03)	0.18 (0.03)	0.18 (0.04)	0.24 (0.06)
Corsi block tapping (*n*_c_ = 20, *n*_BF_ = 19, *n*_AG_ = 14)	Set 4	0.78 (0.04)	0.77 (0.04)	0.78 (0.06)	0.76 (0.05)	0.79 (0.05)	0.83 (0.05)
	Set 5	0.64 (0.05)	0.63 (0.05)	0.58 (0.06)	0.54 (0.06)	0.60 (0.06)	0.61 (0.06)
	Set 6	0.18 (0.05)	0.21 (0.04)	0.19 (0.05)	0.20 (0.05)	0.08 (0.04)	0.15 (0.05)
	Set 7	0.02 (0.01)	0.03 (0.01)	0.04 (0.02)	0.02 (0.01)	0.04 (0.03)	0.02 (0.02)
ECB recognition (*n*_c_ = 20, *n*_BF_ = 20, *n*_AG_ = 14)		12.00 (0.46)	12.55 (0.42)	12.35 (0.46)	12.20 (0.42)	12.29 (0.55)	12.29 (0.51)
Meaningful memory (*n*_c_ = 20, *n*_BF_ = 20, *n*_AG_ = 14)		12.95 (0.97)	13.30 (0.86)	12.70 (0.97)	14.50 (0.86)	14.07 (1.16)	14.07 (1.02)
MSEQ (*n*_c_ = 20, *n*_BF_ = 20, *n*_AG_ = 14)	Average confidence	63 (2)	63 (4)	62 (4)	61 (5)	63 (6)	66 (6)
Flanker (*n*_c_ = 20, *n*_BF_ = 20, *n*_AG_ = 13)	Congruent RT (ms)	622 (23)	599 (20)	681 (20)	637 (24)	632 (26)	602 (31)
	Incongruent RT (ms)	750 (41)	678 (21)	797 (27)	738 (30)	736 (44)	670 (29)
	Congruent accuracy	0.98 (0.01)	0.99 (0.01)	0.94 (0.04)	0.93 (0.05)	0.95 (0.04)	0.93 (0.04)
	Incongruent accuracy	0.86 (0.06)	0.96 (0.02)	0.85 (0.06)	0.90 (0.04)	0.88 (0.06)	0.91 (0.04)
Task switching (*n*_c_ = 19, *n*_BF_ = 20, *n*_AG_ = 13)	Repeat RT (ms)	1175 (45)	1161 (53)	1193 (40)	1219 (47)	1145 (61)	1109 (46)
	Switch RT (ms)	1480 (55)	1453 (77)	1443 (63)	1553 (63)	1347 (75)	1447 (51)
	Repeat accuracy	0.71 (0.05)	0.75 (0.04)	0.78 (0.03)	0.76 (0.04)	0.79 (0.05)	0.79 (0.06)
	Switch accuracy	0.63 (0.05)	0.71 (0.04)	0.70 (0.03)	0.69 (0.04)	0.74 (0.04)	0.74 (0.05)
Raven’s matrices (*n*_c_ = 19, *n*_BF_ = 18, *n*_AG_ = 12)		6.63 (0.78)	7.21 (0.69)	6.00 (0.79)	6.63 (0.78)	7.00 (0.86)	6.08 (0.97)
ECB reasoning (*n*_c_ = 20, *n*_BF_ = 20, *n*_AG_ = 14)		35.45 (1.21)	34.10 (1.27)	34.70 (1.21)	34.50 (1.27)	37.86 (1.45)	34.29 (1.51)
Letter sets (*n*_c_ = 19, *n*_BF_ = 20, *n*_AG_ = 14)		13.74 (1.33)	15.16 (1.51)	14.00 (1.30)	13.30 (1.47)	16.29 (1.55)	14.93 (1.75)
MIDUS (*n*_c_ = 19, *n*_BF_ = 19, *n*_AG_ = 13)	Autonomy	16.05 (1.17)	17.70 (1.23)	16.05 (1.20)	15.95 (1.27)	12.93 (1.40)	11.57 (1.48)
	Env. mastery	15.58 (1.29)	16.47 (1.39)	14.00 (1.26)	14.40 (1.35)	14.71 (1.51)	13.21 (1.62)
	Positive rel.	12.45 (1.16)	12.60 (1.15)	14.65 (1.16)	14.45 (1.15)	12.85 (1.44)	11.85 (1.43)
	Personal growth	15.25 (1.16)	14.75 (1.05)	11.21 (1.19)	11.68 (1.08)	12.93 (1.39)	10.21 (1.26)
	Life purpose	15.32 (1.27)	15.53 (1.32)	14.50 (1.24)	14.85 (1.29)	13.79 (1.48)	12.93 (1.54)
	Self-acceptance	15.70 (1.31)	15.30 (1.22)	13.00 (1.34)	13.32 (1.25)	12.39 (1.62)	10.31 (1.52)

*Standard errors listed within parenthesis*.

*For the analysis of each measure, *n*_c_ = Number of participants included in Control condition, *n*_BF_ = Number of participants included in Brain Fitness condition, *n*_AG_ = Number of participants included in Action Game condition. For MIDUS, participant count reflects minimum number of participants included in the analysis of each subscale analysis*.

**Figure 1:**
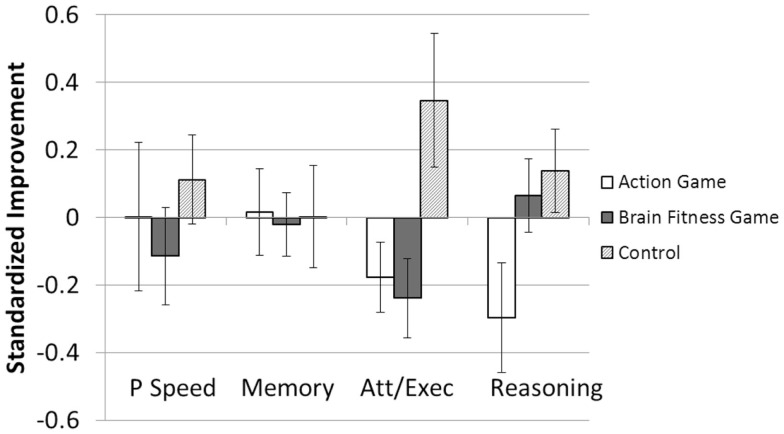
**Standardized (*Z*-score) composite improvement scores as a function or task type and group**. Error bars represent ± SEM.

A number of additional analyses were conducted to search for any hint of a video game effect. For example, it could be that when all measures of performance are considered together rather than individually, a small but general effect of game training is present. To test for this possibility, improvement scores for all objective measures of performance (excluding subjective measures such as MIDUS and the Memory Self-Efficacy Questionnaire) were standardized. These were then averaged across tasks measuring similar constructs to produce composite improvement scores representing Processing Speed (combining Reaction Time, Number Comparison, Visual Search data), Memory (combining Corsi Block Tapping, Everyday Recognition, Meaningful Memory data), Attention/Executive Control (combining Flanker Task and Tasks Switching data), and Reasoning Ability (combining Raven’s Matrices, Everyday Reasoning, and Letter Sets data). Composite measures were entered into an MANOVA with group as a factor and age as a covariate. This indeed revealed an effect of group [*F*(8, 96) = 2.13, *p* < 0.05, $\eta_{p}^{2}$ = 0.15]. While the effect of group was not significant for Processing Speed [*F*(2, 50) = 0.24, *p* = 0.61, $\eta_{p}^{2}$ = 0.02], Memory [*F*(2, 50) = 0.02, *p* = 0.98, $\eta_{p}^{2}$ < 0.01], or Reasoning Ability [*F*(2, 50) = 2.92, *p* = 0.06, $\eta_{p}^{2}$ = 0.11], there was a significant difference between groups on the composite measure of executive control [*F*(2, 50) = 4.36, *p* = 0.02, $\eta_{p}^{2}$ = 0.15]. However, this difference favored the control group rather than the game groups (Figure 1).

### Intervention compliance

Next we explored whether differences in intervention compliance might be responsible for the absence of an action game effect. Recall that participants who received a game intervention were asked to play five times a week for 3 months for a total of approximately 60 h. Based on phone and diary data, we reconstructed the total number of hours played by each participant over the course of the 3-month period^4^. Participants who received the Brain Fitness Game, on average, came very close to the 60 h goal (*M* = 56 h, SD = 6). However, consistent with the hypothesis that older adults would prefer the Brain Fitness Game, participants who received the Action Video Game played for significantly fewer hours [*M* = 22 h, SD = 5, *F*(1, 32) = 8.78, *p* < 0.01, $\eta_{p}^{2}$ = 0.22]. There was no clear relationship between compliance and improvement, although results must be interpreted with caution given the small sample (Table 4; see text footnote 2 for individual task correlations).

**Table 4:** **Correlation coefficients between reported hours of game play and improvement**.

	Brain fitness			Action game		
	*N*	*r*	*p*	*N*	*r*	*p*
Perceptual speed	20	0.28	0.24	14	0.11	0.70
Memory	20	−0.05	0.84	14	0.11	0.70
Attention/executive control	20	0.16	0.49	14	−0.23	0.43
Reasoning	20	−0.33	0.16	14	0.30	0.29

### Attitudes and perceptions

To better understand differences in compliance, we explored data on participants’ attitudes and perceptions of game training. At post-training, participants were given two surveys, one of which focused on their experiences with the game they were given to play, and one which asked them about perceived benefits of game training. Item responses were on a Likert scale, with 1 representing strong disagreement and 7 representing strong agreement with given statements.

#### Perception of game training questionnaire

**Figure 2:**
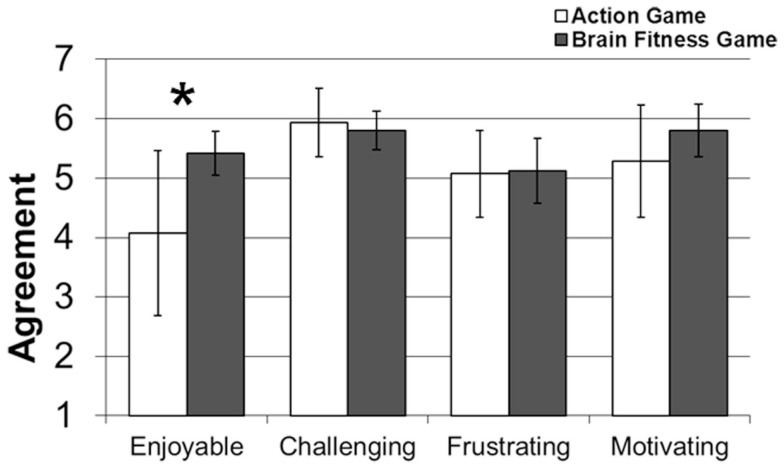
**Game perception agreement scores as a function of game type**. Participants who received the Action Game rated it as significantly less enjoyable. Error bars represent 95% confidence intervals. *p < 0.05.

Participants were asked to rate their agreement with the following statements: (1) I found the game I was given to play *enjoyable*, (2) I found the game I was given to play *challenging*, (3) I found the game I was given to play *frustrating*, and (4) I was *motivated* to perform well on the game I was given to play. The results from the Brain Fitness and Action Game groups are depicted in Figure 2. Scores for each question were entered into an ANOVA, with group as a between-participants factor and question as a within-participant factor^5^. This ANOVA revealed an interaction between group and question [*F*(3, 93) = 2.63, *p* = 0.05, $\eta_{p}^{2}$ = 0.08]. The only question to reveal a significant difference between groups was the question assessing enjoyment. Participants who received the Action Game rated the game as significantly less enjoyable compared to the Brain Fitness Game [*F*(1, 33) = 5.32, *p* < 0.05, $\eta_{p}^{2}$ = 0.15].

#### Perception of video game training effectiveness

**Figure 3:**
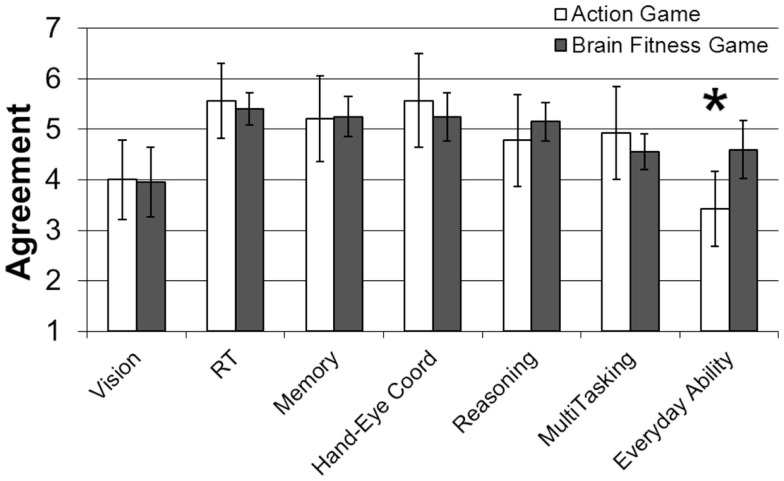
**Perceived benefit agreement scores as a function of game type**. Participants who received the Action Game rated it as significantly less likely to improve everyday abilities. Error bars represent 95% confidence intervals. *p < 0.05.

Participants were asked to rate their agreement with statements in the form of: Video games like the one I was given to play have the potential to improve (1) vision, (2) reaction time, (3) memory, (4) hand-eye coordination, (5) reasoning ability, (6) multi-tasking ability (managing multiple tasks at the same time), (7) the performance of everyday tasks such as driving, remembering important dates, and managing finances. The results from the Brain Fitness and Action Game groups are depicted in Figure 3. An ANOVA revealed an interaction between group and question [*F*(6, 192) = 3.08, *p* < 0.01, $\eta_{p}^{2}$ = 0.08]. The only question to reveal a significant difference between groups was the question regarding everyday abilities. Participants who received the Action Game intervention were significantly less likely to believe the intervention would improve everyday abilities [*F*(1, 32) = 7.20, *p* < 0.05, $\eta_{p}^{2}$ = 0.18].

### Predictors of compliance

Survey data suggested two reasons for the low compliance rate of the Action Game group. First, participants found the game to be less enjoyable. Second, participants were less likely to believe that the game would improve their cognition in a meaningful way. A regression analysis, with compliance as the criterion variable, and game type, enjoyment, and perceived benefit to everyday abilities as predictor variables found that game type was the only significant predictor of compliance [*b* = 30.87, *t*(29) = 2.31, *p* < 0.05]. However, exploratory analyses considering each game group separately found that for the Brain Fitness group, compliance was associated with perceived benefits to reaction time [*r*(20) = 0.63, *p* < 0.01], memory [*r*(20) = 0.51, *p* < 0.05], and hand-eye coordination [*r*(20) = 0.42, *p* = 0.06]. For the Action Game group, perceived benefits were not significantly associated with compliance; however motivation to do well in the game was significantly correlated with perceived benefits to all abilities except vision [*r*(14) > 0.57, *p* values < 0.05]. Game enjoyment in the Action Game group was also significantly correlated with perceived benefits to all abilities except vision [*r*(14) > 0.79, *p* values < 0.05], as was perceived game challenge [*r*(14) > 0.63, *p* values < 0.05]. This pattern of association between perceived benefits and game enjoyment, motivation, and challenge was not observed in the Brain Fitness group. Although exploratory, these results suggest that perceived benefits may play multiple roles in shaping older adults’ attitudes and perceptions of game training.

### Qualitative data

Participants were given the opportunity to make comments about their game experience in the diary they were asked to keep. Comments generally mirrored survey data, with more positive comments related to the Brain Fitness Game compared to the Action Game (Table 5). Although participants generally liked the Brain Fitness game, some problems were noted, especially with the text and speech recognition functions of the game. Participants were frustrated in instances in which they knew the correct answer, but were marked as being incorrect because the game did not recognize what they said or wrote. Compared to the Brian Fitness Game, participants in the Action Game Group reported more problems and frustration, including difficulties interacting with the game due to arthritis and eyestrain. A number of participants explicitly noted a lack of interest in content of the game.

**Table 5:** **Representative positive and negative quotes regarding training**.

**POSITIVE BRAIN FITNESS QUOTES**
“Feel good about decreasing brain age.” – Participant A (Female, Age 78)
“I do all the games, I am doing them faster.” – Participant B (Female, Age 70)
“Enjoying the games but not good at many of them. I like the piano, but not a ‘true pianist’ yet.” – Participant C (Female, Age 70)
“This has been fascinating- wish I could improve, going to try in the AM.” – Participant D (Female, Age 75)
“I’m addicted!! What am I going to do when this test is done? Go buy a game? Steal this one? Or tell my son I need one?” – Participant E (Female, Age 69)
**NEGATIVE BRAIN FITNESS QUOTES**
“The software makes more mistakes than I do.” – Participant B (Female, Age 70)
“Game does not always show the numbers I want to write.” – Participant A (Female, Age 78)
“Still problems with machine reading correctly – kills competitive spirit.” – Participant F (Male, Age 71)
“It is frustrating to get a correct answer and have it misread!” – Participant G (Male, Age 68)
“Barking dogs can ruin rock, paper, scissors.” (referring to a game involving voice recognition) – Participant H (Female, Age 79)
**POSITIVE ACTION GAME QUOTES**
“Did time trials, competitive nature taking over.” – Participant I (Male, Age 75)
“Used booklet to note characteristics of drivers-enjoyable, more interested.” – Participant J (Male, Age 80)
“Actually enjoyed it. It went very well. Many 1st places.” – Participant K (Female, Age 78)
**NEGATIVE ACTION GAME QUOTES**
“Noticing eye strain after 30 minutes.” – Participant L (Female, Age 66)
“I have arthritis in my hands. When I play more than 30 minutes it really hurts but I am trying.” – Participant M (Female, Age 69)
“Awkward! Re-read manual and try[ing] to coordinate actions. Arthritis in hands makes some action uncomfortable.” – Participant N (Male, Age 86)
“Mindless; challenge is dexterity rather than thinking. Utterly boring.” – Participant I (Male, Age, 75)
“Running a little guy around a race track is inherently less interesting than reading, movies, or computer games like free cell, hearts, or black jack.” – Participant O (Male, Age 66)

## Discussion

Previous studies have found that relatively short action video game interventions can result in dramatic improvements to a number of perceptual and cognitive abilities (but see also Boot et al., 2008, 2011). Thus video game interventions are potentially an ideal solution to address the many perceptual and cognitive declines associated with aging. Basak et al. (2008) found that in an older adult sample, a video game intervention was capable of improving memory, executive functioning, and reasoning ability. The current study built upon this prior work to examine the effectiveness of an action game intervention compared to a brain fitness game intervention and found that neither resulted in greater cognitive improvement compared to a no-game control group.

While on the surface results are disappointing, the lack of action game effect must be viewed in the context of low compliance and negative attitudes toward the game predicted to induce the largest improvements. Low intervention compliance was consistent with older gamers’ preference for intellectually challenging games over games that require quick reflexes and fast reaction time (Pearce, 2008). Participants rated the action game as significantly less enjoyable compared to the brain fitness game, and did not believe the action game had the potential to improve important everyday abilities such as driving.

Additional study limitations are worth discussing. Within each game, participants had many options from which to choose. In *Mario Kart*, participants could choose any level of difficulty they felt comfortable with, concentrate on a few race tracks and racers, or explore diverse race tracks and play many different characters. In *Brain Age*, participants could play Sudoku or engage in either a few or many diverse game activities with different demands. Relatively unconstrained (but externally valid) training in which participants were free to choose activities within each game, and how long to spend on each activity, may have contributed to null results. Furthermore, given this freedom, it was impossible to compute meaningful learning curves for participants’ game performance. Thus, we cannot compare amount of improvement in game to the amount of transfer observed. If some participants demonstrated no-game improvement it is unlikely they would demonstrate transfer. Additionally, the largest effects in the literature have been found with action game training, mostly training on first-person shooters (e.g., Green and Bavelier, 2003, 2006a,b). There could be important differences between these games and the racing game *Mario Kart*, which might explain a lack of effect (such as the degree to which peripheral monitoring is necessary). There are likely important game elements (such as the degree to which task switching is required) that differ between *Mario Kart* and the more strategic game used by Basak et al. (2008). Finally, the seniors in our study were relatively cognitively intact (with a high average MMSE score) and well-educated. Training may be more effective for individuals who are more impaired.

It should be noted that both groups tended to agree that the game they were given to play was frustrating (Figure 2). For the *Brain Age 2*^™^ game in particular, this frustration appears to stem partly from the game’s use of handwriting recognition. Participants almost universally expressed some degree of frustration with this aspect of the game. For the *Mario Kart DS*^®^ game, arthritis-related pain and eyestrain were reported by some participants. It is not particularly surprising that the this group reported more arthritis-related problems since the game system had to be held in such a way that the system was supported with the fingers of each hand, while the *Brain Age 2*^™^ game allowed participants to hold the system in the palm of one hand. The *Brain Age 2*^™^ interface was navigated almost exclusively with a stylus and touch screen, while *Mario Kart DS*^®^ required using a directional pad and game buttons. A focus on ergonomics and human factors, especially with respect to the needs of the older adult user, may make technology-based cognitive interventions more accessible and enjoyable for older adults (Charness and Boot, 2009; Boot et al., 2012).

Our results contrast with those of Nouchi et al. (2012), who found broad improvements as a result of *Brain Age 2*^™^ training after only 5 h of gameplay (15 min of gameplay 5 days a week for 4 weeks). Our intervention was rather long. On average, participants in our Brain Fitness group played the same game for more than 50 h, yet no evidence of transfer was observed. Another recent study found transfer (but not far transfer) as a result of online brain-training (van Muijden et al., 2012). At this point the reason for conflicting results remains uncertain. Different assessment tasks used to measure cognition may be one explanation. Our results were more consistent with those of Ackerman et al. (2010) and Owen et al. (2010).

In sum, video game interventions may hold promise in terms of addressing declines associated with cognitive aging, but there are still many unknowns. A greater understanding of the mechanisms underlying general transfer induced by action video game play needs to be a major goal of this line of research, but is a particularly challenging problem given the complexity of modern action video games. Once isolated, the key components of what make action games so successful in terms of improving general abilities might be embedded within games more appealing to older adults. We found that a belief that an intervention is capable of improving abilities was associated with increased compliance, and this information might be incorporated into new video game interventions. Finally, researchers must recognize individual differences in game preference. Among younger adults, not all players enjoy the same type of game experience, and the same is true of older adults. The most successful cognitive intervention in the world is essentially worthless unless individuals are willing and able to engage in it. Thus efforts need to be made not just to understand what interventions are capable of improving cognition, but how to structure and deliver these interventions to ensure that people engage in them.

## Conflict of Interest Statement

The authors declare that the research was conducted in the absence of any commercial or financial relationships that could be construed as a potential conflict of interest.

## Footnotes

^1^Note that this particular measure of task-switch cost may have put us at a disadvantage to detect an effect, the largest age-related switch costs are observed in the difference between single-task blocks and dual-task blocks of trials (i.e., general rather than specific switch costs; Kray and Lindenberger, 2000; Reimers and Maylor, 2005).

^2^http://walterboot.net/GameStudy/GameTrainingQuest.pdf

^3^http://walterboot.net/GameStudy/AnalysisSupplement.pdf

^4^Although we did not obtain objective measures of intervention compliance, it should be noted that each method of determining compliance (diary and phone) produced consistent estimates (Cronbach’s α = 0.93). This gives us confidence that compliance measures were reliable and valid. Compliance analyses used the average compliance assessed by each measure.

^5^One participant in the Brain Fitness Group failed to answer one question, thus their data was not included in the ANOVA, but was included in follow-up contrasts involving the other three questions.
